# Rapidly fluctuating environments constrain coevolutionary arms races by impeding selective sweeps

**DOI:** 10.1098/rspb.2013.0937

**Published:** 2013-08-07

**Authors:** Ellie Harrison, Anna-Liisa Laine, Mikael Hietala, Michael A. Brockhurst

**Affiliations:** 1 Department of Biology, University of York, York YO10 5DD, UK; 2 Metapopulation Research Group, Department of Biosciences, University of Helsinki, PO Box 65, 00014, Finland

**Keywords:** host–parasite, exploiter–victim, natural enemy, arms race, experimental evolution, antagonistic coevolution

## Abstract

Although pervasive, the impact of temporal environmental heterogeneity on coevolutionary processes is poorly understood. Productivity is a key temporally heterogeneous variable, and increasing productivity has been shown to increase rates of antagonistic arms race coevolution, and lead to the evolution of more broadly resistant hosts and more broadly infectious parasites. We investigated the effects of the grain of environmental heterogeneity, in terms of fluctuations in productivity, on bacteria–phage coevolution. Our findings demonstrate that environmental heterogeneity could constrain antagonistic coevolution, but that its effect was dependent upon the grain of heterogeneity, such that both the rate and extent of coevolution were most strongly limited in fine-grained, rapidly fluctuating heterogeneous environments. We further demonstrate that rapid environmental fluctuations were likely to have impeded selective sweeps of resistance alleles, which occurred over longer durations than the fastest, but not the slowest, frequency of fluctuations used. Taken together our results suggest that fine-grained environmental heterogeneity constrained the coevolutionary arms race by impeding selective sweeps.

## 1. Introduction

The importance of environmental heterogeneity for antagonistic species interactions was recognized over 50 years ago by the ‘disease triangle’ concept, which identifies host genotype, pathogen genotype and the environment as the primary determinants of infection outcome [1]. Despite this conceptual advance, until recently studies of antagonistic coevolution have often treated environmental heterogeneity as ‘noise’ that has been excluded from experimental work and theoretical models [2]. However, the geographic mosaic theory of coevolution has refocused attention on how the environment, both abiotic and biotic, may alter the rate and direction of coevolutionary dynamics through genotype × (genotype ×) environment interactions generating selection mosaics across landscapes as the environment varies [3]. In support of this, empirical studies across a range of different biological systems have demonstrated that important facets of antagonistic species interactions are environmentally mediated [2,4], including host resistance, costs of resistance, parasite infectivity, parasite latency, transmission and virulence [5–9].

A key component of environmental heterogeneity in natural populations is variation in productivity [10]. Theoretical and empirical studies suggest that increasing productivity acts to intensify antagonistic coevolution [11–13]. This arises, in part, because increasing productivity tends to increase victim, and thereby exploiter, population sizes [11]. This has two effects: first, it increases victim–exploiter encounter rates, thereby intensifying reciprocal selection [12,14]; second, it increases the supply of mutations, which can affect both the quantity and quality of beneficial mutations available to reciprocal selection [12,15]. In addition, for coevolutionary arms races, increasing environmental productivity can reduce the relative cost to victims of defence mutations, favouring the evolution of costly defence, which in turn increases selection for the evolution of exploiter counter-defence [11,12]. Taken together, these factors lead to accelerated coevolutionary dynamics, and potentially greater escalation of defence and counter-defence traits at the interface of victim–exploiter interaction in more productive environments [11,12]. Moreover, because qualitatively different mutations can be favoured under different productivities (as a result of variation in mutation supply and associated costs), differences in environmental productivity can alter the trajectory of antagonistic coevolution [15].

Environmental productivity can vary in both spatial and temporal dimensions; however, these forms of heterogeneity are unlikely to have equivalent effects on coevolutionary processes [16]. Where productivity is spatially heterogeneous, and distinct subpopulations experience different levels of environmental productivity, local coevolutionary processes can be influenced by immigrating genotypes selected under contrasting productivities. Thus, under spatial heterogeneity, even low rates of gene-flow across productivity gradients can favour genotypes from high-productivity populations in low-productivity patches, where they would otherwise not be observed [11]. This acts to increase coevolutionary rates in low-productivity populations to levels similar to those in high-productivity populations [17–19]. In effect, high-productivity populations act as spatial refuges, providing emigrating genotypes that set the pace of coevolution across the entire landscape [20,21]. Populations that experience temporal fluctuations in environmental productivity will, by contrast, lack refuges to maintain interacting genotypes maladapted to the prevailing environment [22,23]. Periods of low productivity are likely to select against highly costly resistance mutations and, through reducing population sizes, weaken reciprocal selection and lead to higher rates of stochastic loss of rare beneficial genotypes; combined, these effects suggest that fluctuations in productivity may constrain antagonistic arms race coevolution by impeding the inherent recurrent selective sweeps. In support of this, recent empirical findings suggest that resource pulses constrained the evolution of defence by *Serratia marcescens* against its protist predator *Tetrahymena thermophila* relative to populations cultured in constant resource environments [24].

Theory predicts that the frequency, or grain, of environmental fluctuations is an important determinant of their effect on antagonistic coevolution [13,25]. Specifically, in a host–parasite model where environmental fluctuations mediated the strength of reciprocal selection and the specificity of host–parasite interaction, the strength of selection declined, relative to constant environments, with increasing speed of the environmental fluctuations [25]. Similarly, in a victim–exploiter model, investment in victim defence and exploiter attack traits increased with increasing duration of the period of high productivity [13]. We hypothesized therefore that (i) fluctuations in environmental productivity would constrain antagonistic arms race coevolution, and (ii) this effect would be stronger in more rapidly fluctuating environments.

We tested our hypotheses by experimental coevolution of laboratory populations of the bacterium *Pseudomonas fluorescens* and its naturally associated phage *Φ*2 [26,27]. The coevolutionary dynamics of this antagonistic species interaction are well studied [28]; during the early stages of coevolution (i.e. those studied here), coevolution proceeds as an arms race with predominantly directional selection favouring recurrent selective sweeps and escalation of bacterial resistance and phage infectivity traits [29]. Consistent with theoretical assumptions, more productive environments support higher population densities and reduced costs of bacterial resistance [12,15]; moreover, increasing productivity is known to allow increased rates of coevolution, as well as the evolution of more broadly resistant bacteria and more broadly infectious phages [12]. Replicate populations of *P. fluorescens* and phage *Φ*2 were propagated under either temporally homogeneous (constant) or temporally heterogeneous (fluctuating) productivity environments. We manipulated environmental fluctuations in nutrient availability to the host by serially transferring bacteria–phage populations between high- and low-productivity environments at three grains of environmental heterogeneity. We also propagated control populations at the mean nutrient level of the fluctuating treatments. In addition, to confirm the effect of our nutrient manipulation *per se* on coevolutionary dynamics, we propagated populations at the constituent nutrient levels. For each population, we characterized the dynamics and outcomes of arms race coevolution. Furthermore, to determine whether environmental fluctuations would have interfered with the dynamics of selection, we characterized the effect of productivity on the time scale of selective sweeps of bacterial resistance mutations.

## 2. Material and methods

### (a) Culture techniques

Cultures were grown in microcosms, which were 30 ml glass universal bottles with loose-fitting plastic caps containing 6 ml of culture medium with either high, medium or low nutrient levels depending upon treatment. Specifically, three nutrient levels were obtained by serial dilution of standard Kings' B (KB) broth into M-9 salt solution; nutrient concentrations were as follows: high = 1 × standard KB; medium = 0.55 × standard KB; low = 0.1 × standard KB. This range of media concentrations was selected because of known effects on bacterial density and costs of resistance: 1 × KB supports approximately twofold higher bacterial density than 0.1 × KB [12,15]; selection against bacterial resistance mutations in the absence of phages is stronger in 0.1 × KB than in 1 × KB [12] (these patterns were independently verified for this study; see the electronic supplementary material). Cultures were incubated statically at 28°C and propagated by serial transfer whereby 1 per cent of each culture was subcultured into a fresh microcosm every 48 h. Samples of cultures were stored every fourth transfer at −80°C in 20 per cent glycerol. Phage populations were isolated every fourth transfer by centrifuging samples of culture in 10 per cent chloroform to lyse and pellet bacterial debris, and then stored at 4°C.

### (b) Experimental design

Six independent colonies of *P. fluorescens* SBW25 (henceforth ‘independent clones’) were isolated on KB agar and grown overnight in separate microcosms at 28°C shaken at 200 r.p.m. Each independent clone was then used to found one replicate population within each treatment. Specifically, for each independent clone, six populations were founded with 10^7^ *P. fluorescens* SBW25 cells and 10^5^ viral particles from a refrigerated stock of phage previously grown from an individual plaque. One of these populations was assigned to each of the following treatments: alternating 1 × KB and 0.1 × KB every transfer (fine-grained heterogeneous environment), alternating 1 × KB and 0.1 × KB every two transfers (medium-grained heterogeneous environment), alternating 1 × KB and 0.1 × KB every four transfers (coarse-grained heterogeneous environment) and constant 0.55 × KB (homogeneous environment). In addition, one population from each independent clone was assigned to each of the following constituent productivity treatments: constant 0.1 × KB (low productivity) and constant 1 × KB (high productivity). Each transfer corresponds to approximately 7.5 bacterial generations. Populations were propagated for 16 transfers.

### (c) Quantifying resistance and infectivity

Bacterial resistance was assayed as a binary trait, such that a given bacterial colony could be either susceptible or non-susceptible to infection by phage. For each assayed population (detailed later), 10 individual bacterial colonies were isolated by plating on a KB agar plate. Ten evolved colonies and a colony of the ancestral genotype were then streaked across a 20 μl line of phage on a KB agar plate and incubated for 24 h at 28°C. A colony was defined as susceptible if there was visible inhibition of growth upon crossing the line of phage. Resistance was recorded as the proportion of non-susceptible bacteria per population, while infectivity was measured as the proportion of susceptible bacteria per population [14,26]. The nutrient level of the KB agar plate test environment did not affect the proportion of resistant colonies observed (data not shown).

### (d) Time-shift assay

The rate of coevolution is the product of host and parasite evolutionary rates. Therefore to estimate the rate of coevolution, we used stored population samples to measure both (i) the rate of phage infectivity evolution (i.e. how does the infectivity of phage populations to a bacterial population change through time?) and (ii) the rate of bacterial resistance evolution (i.e. how does the resistance of bacterial populations to a phage population change through time?). Specifically, at transfers 8 and 12, we determined (i) the infectivity (proportion susceptible colonies) of past (four transfers previous), contemporary and future sympatric phage populations against a given bacterial population, and (ii) the resistance (proportion resistant colonies) of past (four transfers previous), contemporary and future sympatric bacterial populations against a given phage population. The rate of directional trait evolution is proportional to the slope of phage infectivity or bacterial resistance over the time-shift [14]. If directional antagonistic coevolution was occurring then, for both infectivity and resistance, we would expect positive slopes against time-shift (i.e. for infectivity, future phage would be expected to be better than contemporary phage, and contemporary phage better than past phage, at infecting contemporary bacteria [14]).

### (e) Cross-infection assay

To determine the relative extent of coevolution in each treatment, we performed a cross-infection assay across treatments. The breadth of resistance range and infectivity range was assayed every four transfers by determining the resistance/infectivity for each bacteria–phage population when assayed against populations founded from the same independent clone from all treatments and at the same timepoint. This provides, at each assayed timepoint, a ‘global’ measure of which treatment has produced the relatively most infectious and resistant populations [30,31].

### (f) Tracking selective sweeps of resistance alleles

It is likely that an important determinant of the effect of environmental fluctuations on coevolution is the relative time scales of environmental fluctuations and selective sweeps of beneficial mutations. One way to quantify the time scale of a selective sweep in a bacterial population is to monitor deviations in the frequency of genetic markers—sharp deviations in marker frequency suggest that either the marked or unmarked background has become linked to a beneficial mutation on its way to fixation [32]. In the presence of phage, this therefore allows us to track the progress of the first selective sweep of bacterial resistance. Populations were founded with approximately equal proportions of *P. fluorescens* SBW25 and SBW25-*lacZ*, an isogenic marked strain carrying a *lacZ* insertion, which appears blue on KB media supplemented with X-gal [33]. Selective sweeps were defined as when either marked or unmarked colonies reached more than 99 per cent of the population. We employed a full-factorial experimental design with two levels of productivity (0.1 × KB or 1 × KB) and two levels of phage (present or absent), giving a total of four treatments. Twelve replicate populations were assigned to each treatment (total 48 populations) and were propagated for 10 serial transfers under conditions identical to those described for the main selection experiment. At every transfer, we estimated bacterial density and the proportion of each marker type by plating serial dilutions of each population onto KB agar plates supplemented with 50 µg ml^−1^ of X-gal, at a density of approximately 100–500 colonies per plate. Phage densities were also estimated at every transfer by spotting serial dilutions of phage population samples onto exponentially growing lawns of SBW25 in soft-agar overlays on KB agar plates.

### (g) Statistical analyses

Infectivity and resistance data were analysed by repeated measures linear mixed-effects models fitted by restricted maximum likelihood in JMP v. 10. To test the effect of treatments on the rate of coevolution, resistance and infectivity data from the time-shift assays were analysed in separate models fitting ‘treatment’ (categorical variable, coding either ‘productivity’ or ‘grain’ treatments), ‘timepoint’ and ‘time-shift’ (covariates), and their interactions as fixed effects, and ‘founding clone’ and ‘population’ nested within ‘founding clone’ as random effects. (Note that ‘time-shift’ was fitted as a linear covariate because we were interested here in detecting change in the rate of trait evolution in response to directional selection.) To test the effect of treatments on the extent of coevolution, resistance range and infectivity range data from the cross-infection assay were analysed in separate models fitting ‘treatment’, ‘timepoint’ and their interaction as fixed effects, and ‘founding clone’ and ‘population’ nested within ‘founding clone’ as random effects. Separate analyses were performed to test for (i) the effect of productivity *per se* on the rate and extent of coevolution (i.e. by comparing the high- and low-productivity treatments), and (ii) the effect of the grain of environmental heterogeneity on the rate and extent of coevolution (i.e. by comparing the constant 0.55 × KB homogeneous and heterogeneous environment treatments). Because of the sequence of environmental alternations, the coarse-grained heterogeneous treatment (i.e. alternating every fourth transfer) had experienced a higher level of cumulative productivity than had the other treatments at the fourth and twelfth transfer (i.e. the actual resources supplied to these populations at these timepoints was higher than would be expected if resource supplies were equal across treatments). To control for this, models testing the effect of the grain of environmental heterogeneity included an additional covariate, ‘resource ratio’, which was calculated as ratio of actual to expected resource supply experienced by the focal bacterial or phage population at that timepoint. To conform to model assumptions (i.e. normality, homogeneity of variance), infectivity and resistance data were arcsine-square-root-transformed, infectivity range data were arsine-transformed and resistance range data were square-root-transformed.

## 3. Results

### (a) Rates of evolution of resistance and infectivity traits

**Figure 1.**
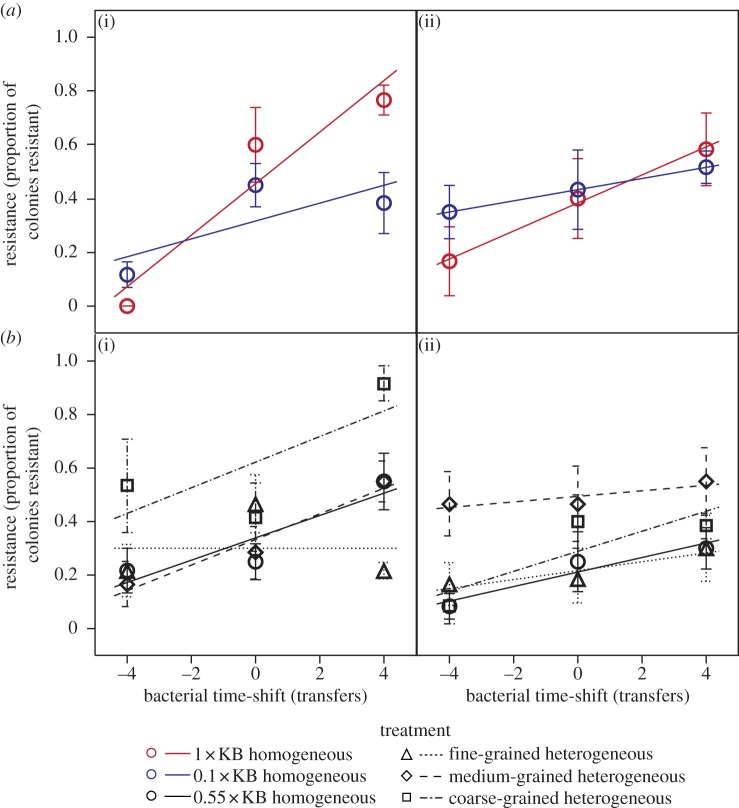
Time-shift of bacterial resistance. Effects of (*a*) productivity and (*b*) the grain of environmental heterogeneity on the rate of bacterial resistance evolution. Data points show mean resistance of past, contemporary and future bacterial populations against phage populations from transfers (i) 8 and (ii) 12; error bars denote ±1 s.e.; the slopes of regression lines are proportional to the rate of bacterial resistance evolution.

**Figure 2.**
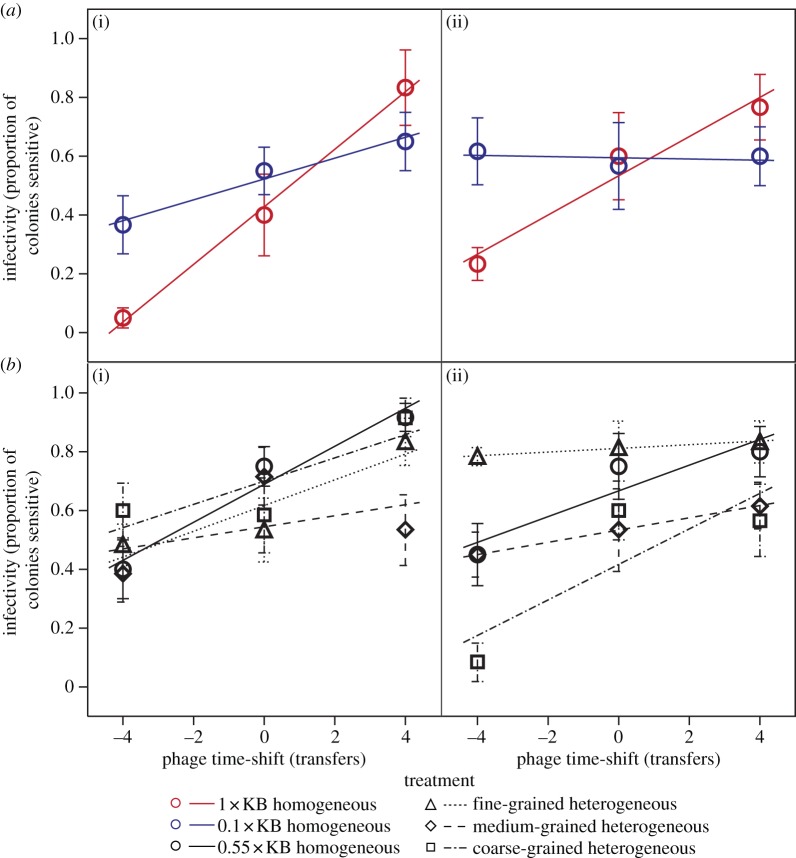
Time-shift of phage infectivity. Effects of (*a*) productivity and (*b*) the grain of environmental heterogeneity on the rate of phage infectivity evolution. Data points show mean infectivity of past, contemporary and future phage populations against bacterial populations from transfers (i) 8 and (ii) 12; error bars denote ±1 s.e.; the slopes of regression lines are proportional to the rate of phage infectivity evolution.

Among the constituent homogeneous environments, increasing productivity accelerated the rate of both infectivity and resistance evolution (figure 1*a*, resistance: productivity × time-shift interaction, *F*_1,54_ = 10.52, *p* = 0.002; figure 2*a*, infectivity: productivity × time-shift interaction, *F*_1,54_ = 16.48, *p* = 0.0002), although rates of infectivity evolution declined over time in both treatments (cf. transfers 8 and 12; figure 2*a*, timepoint × time-shift interaction, *F*_1,54_ = 5.38, *p* = 0.024). This confirms that our productivity manipulation significantly altered baseline coevolutionary dynamics as anticipated.

The grain of environmental heterogeneity significantly altered the rate of evolution of both resistance and infectivity traits (figure 1*b*, resistance rate: grain × time-shift interaction, *F*_3,107_ = 3.07, *p* = 0.031; figure 2*b*, infectivity rate: grain × time-shift interaction, *F*_3,107_ = 4.46, *p* = 0.0054). For resistance traits, the rate of evolution was significantly lower in the fine-grained environment compared with both the coarse-grained and homogeneous environments, and higher in coarse-grained compared with the homogeneous environment (within model contrasts, all *p* < 0.05). For infectivity traits, the rate of evolution was higher in the coarse-grained environment compared with the other heterogeneous environments and the homogeneous environment, and lower in the medium-grained heterogeneous environment compared with the homogeneous environment (within model contrasts, all *p* < 0.05). Together, this suggests that frequent exposure to low productivity decelerated coevolution in more rapidly fluctuating, finer-grained heterogeneous environments.

### (b) Extent of escalation of resistance and infectivity ranges

**Figure 3.**
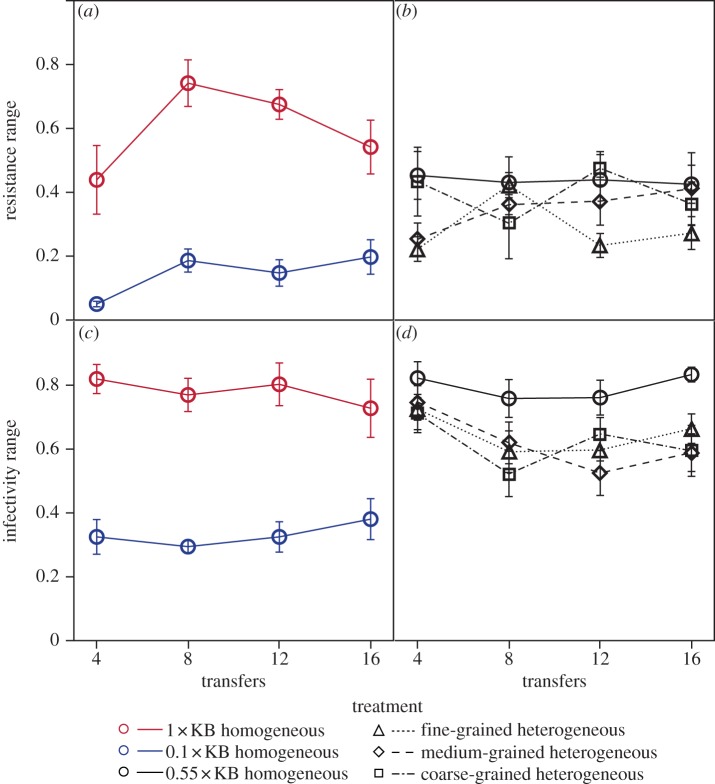
The ranges of bacterial resistance and phage infectivity. Effects of (*a*,*c*) productivity and (*b*,*d*) the grain of environmental heterogeneity on the breadth of (*a*,*b*) bacterial resistance range and (*c*,*d*) phage infectivity range through time. Data points show mean bacterial resistance range or mean phage infectivity range at a given timepoint; error bars denote ±1 s.e.

Among the constituent homogeneous environments, increasing productivity increased the breadths of bacterial resistance and phage infectivity ranges that evolved (figure 3*a*, resistance range: treatment, *F*_1,5_ = 93.89, *p* = 0.0002; figure 3*c*, infectivity range: treatment, *F*_1,5_ = 52.97, *p* = 0.0008), confirming that our productivity manipulation significantly altered the extent of evolutionary escalation of these traits as anticipated.

The breadth of evolved bacterial resistance range varied with the grain of environmental heterogeneity: broader resistance ranges evolved in the homogeneous environment, while the narrowest resistance ranges evolved in the fine-grained heterogeneous environment (figure 3*b*, treatment, *F*_3,20.07_ = 3.09, *p* = 0.0503). Similarly, phages evolved broader infectivity ranges in the homogeneous environment than in the heterogeneous environments (figure 3*d*, treatment, *F*_3,19.85_ = 8.92, *p* = 0.0006). Together this suggests that frequent exposure to low productivity constrained the evolution of broad bacterial resistance ranges, and that exposure to low productivity *per se*, irrespective of the frequency of exposure, limited phage infectivity range evolution.

### (c) Effects of productivity on the time scale of selective sweeps

**Figure 4.**
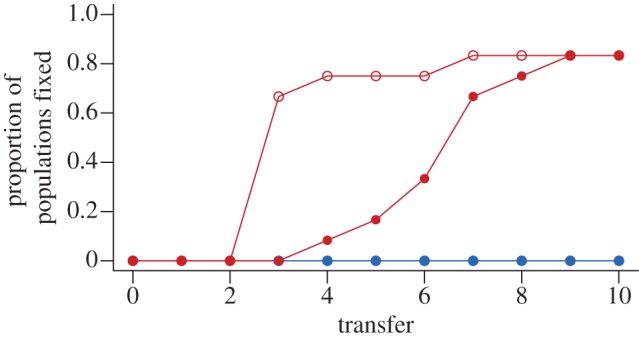
The dynamics of selective sweeps. Effects of productivity and phage on selective sweeps. Data points show the proportion of populations in which either the unmarked or *lacZ*-marked strain has reached a frequency of greater than 99% though time. Populations grown in the 1 × KB environment are shown in red and those grown in the 0.1 × KB environment are shown in blue. Open circles denote phage-containing populations, and closed circles denote phage-free populations.

Over the course of 10 transfers, selective sweeps were not observed in the low-productivity environment irrespective of the presence or the absence of phage. By contrast, selective sweeps were observed in the majority of populations in the high-productivity environment, both in the presence and absence of phage. The time scale of selective sweeps under high productivity was significantly faster in the presence of phage (figure 4; without phage = 6.6 transfers ± 0.45 s.e.; with phage = 3.5 transfers ± 0.40 s.e.; Welch's *t*-test, *t*_17.75_ = −5.127, *p* < 0.0001). While selective sweeps in the absence of phage were always associated with loss of the marked strain, in the presence of phage, selective sweeps occurred in both the unmarked and marked genetic backgrounds. This suggests that in the absence of phage, sweeps were due solely to selection against the marker, presumably because of costs associated with the *lacZ* gene insertion. Contrastingly, in the presence of phage, the faster sweeps were likely to have been caused by linkage of the marked or unmarked genetic backgrounds to resistance mutations. These data therefore suggest that populations in coarse-grained heterogeneous environments could indeed undergo completed selective sweeps during periods of exposure to high productivity, and, concomitantly, that exposure to low productivity impedes selective sweeps (figure 4).

## 4. Discussion

Our findings demonstrate that environmental heterogeneity, constituting fluctuation between high and low productivity, can act to decelerate antagonistic coevolution, and constrain evolutionary escalation in the breadth of bacterial resistance range and phage infectivity range. Moreover, we show that these patterns are dependent upon the speed of environmental fluctuation, such that coevolution is constrained most strongly in fine-grained, rapidly fluctuating heterogeneous environments. Theory suggests that low-productivity environments weaken reciprocal selection through reduced encounter rates, reduce the supply of host resistance mutations and exacerbate the costs associated with such mutations [11,12]. In support of this, bacterial densities were approximately twofold lower in 0.1 × media compared with 1 × media (see electronic supplementary material, figure S1; but see also [12,15]). Moreover, this study (see electronic supplementary material, figure S2) and previous studies with this system have demonstrated that selection against bacterial resistance mutations in the absence of phage is stronger in 0.1 × media compared with 1 × media [12]. Therefore, periodic exposure to low productivity is likely to have impeded both the emergence and maintenance of bacterial resistance mutations, retarding the evolution of bacterial resistance, and thereby weakening selection for the evolution of phage infectivity.

Why then is antagonistic coevolution constrained to a greater degree by faster environmental fluctuations? Under high productivity, coevolution in this system is thought to proceed, for approximately the first 200 generations, through a series of recurrent selective sweeps of resistance and infectivity alleles [29,34,35]. A possible explanation, therefore, is that rapid fluctuations occur on shorter time scales than selective sweeps, and thus rapid fluctuations could impede the rise in frequency of resistance and infectivity alleles. Incomplete sweeps would be compounded by intervening periods of low productivity, which would strongly select against costly resistance alleles. By contrast, if environmental fluctuations occur over longer (or similar) time scales to selective sweeps, these would allow resistance and infectivity alleles to rise to high frequency during high-productivity periods. High-frequency resistance alleles would then be less prone to loss, either through stochastic loss or purifying selection, during intervening low-productivity periods. Consistent with this, we observed that selective sweeps of markers linked to resistance mutations took approximately 3.5 transfers in high-productivity environments, but contrastingly were not observed under low productivity. This suggests that selective sweeps can occur under high productivity on time scales shorter than the frequency of fluctuations in our coarse-grained heterogeneous environment. Rapid environmental fluctuations, occurring every transfer, would have been likely to interfere with these selection dynamics.

These data are consistent with recent theory predicting slower coevolutionary dynamics in populations experiencing more rapid environmental fluctuations [25]. In this model, the effects of environmental fluctuations on selection are decomposed into short-term and long-term effects: short-term effects stem from changes in selection coefficients from one generation to another, whereas long-term effects stem from the average selection coefficients over many generations. When environments fluctuate rapidly, long-term selection acting on populations is weaker than in constant environments, whereas under slower environmental fluctuations short-term and long-term effects merge, resulting in dynamics similar to those observed in constant environments. In their model, Mostowy & Engelstädter [25] directly impose temporal heterogeneity in the strength and specificity of selection. By contrast, we manipulated an abiotic variable, environmental productivity, which nonetheless is likely to have affected both of these properties of selection. We confirm the previous finding that increasing resource supply accelerates coevolution [12], which is likely to be due, at least in part, to intensification of reciprocal selection. Moreover, heterogeneity in environmental productivity between populations has been shown previously in this system to drive greater phage local adaptation, suggesting that different productivity regimes cause divergent coevolutionary trajectories, favouring distinct resistance and infectivity specificity phenotypes under different levels of productivity [15]. Unfortunately, however, it is difficult to draw direct comparisons between our findings and this model, since this experimental system does not conform to either of the forms of infection genetics employed (i.e. matching alleles or gene-for-gene).

It is valuable to contrast our findings with those of earlier studies of the effects of pulsed resource supply dynamics on coevolution of the bacterium *S. marscesens* and the protist predator *T. thermophila* [24,36,37] (although note that Hiltunen *et al*. [37] employ a community of prey bacteria of which *S. marscesens* is one of the constituent species, and thus is less readily comparable with our study). These experiments reveal inconsistent effects of resource pulses on coevolution: Friman & Laakso [24] reported that antagonistic coevolution was constrained in pulsed resource environments relative to constant environments, whereas Friman *et al*. [36] reported no effect of resource pulses on mean coevolutionary changes. Interestingly, these experiments used different grains of environmental heterogeneity; specifically, high-productivity pulses occurred for 1 day in every 7 days [24], or for 5 days in every 10 days [36], corresponding to relatively finer- and coarser-grained resource pulses, respectively. Our finding that antagonistic coevolution was most strongly constrained in fine-grained heterogeneous environments may therefore help to explain the inconsistent effects of resource pulses on coevolution between *S. marscesens* and *T. thermophila*.

Friman & Laakso [24] observed strong effects of resource pulses on *T. thermophila* population dynamics, which peaked following resource pulses before rapidly declining to population densities lower than those observed in constant environments. Overall, these ecological dynamics appear to have reduced the strength of selection on *S. marscesens* to evolve resistance to predation [24]. Although we did not explicitly quantify ecological dynamics in our experimental populations, we did determine the effects of high and low productivity on the densities of bacteria and phage (see electronic supplementary material). While bacterial densities were higher in high-productivity compared with low-productivity environments (see electronic supplementary material, figure S1*a*,*c*), surprisingly, phage densities were unaffected by environmental productivity (see electronic supplementary material, figure S1*b*,*d*). This suggests that, in contrast to the *S. marscesens*–*T. thermophila* experiments, our findings are unlikely to have been caused by large resource-driven fluctuations in exploiter density.

Most theoretical and experimental work on the effects of environmental heterogeneity on coevolution has focused on spatial abiotic [11,17–21,38] rather than temporal abiotic heterogeneity (although see [16,24,36,37,39]). Our findings suggest that these contrasting forms of environmental heterogeneity are unlikely to be equivalent in antagonistic coevolving systems. In particular, frequent exposure of populations in rapidly fluctuating environments to low productivity prevented these populations from attaining coevolutionary dynamics commensurate with those observed in the constant high-productivity homogeneous environment. In addition, the extent of escalation of bacterial resistance range and phage infectivity range in heterogeneous environments was intermediate between that observed in the constant low-productivity and high-productivity homogeneous treatments. This confirms our prediction that, owing to the lack of spatial refuges, temporal heterogeneity would not recapitulate the pacemaker effects observed in spatially heterogeneous coevolving bacteria–phage populations with gene-flow, whereby landscape-level coevolutionary rates are set by the fastest-coevolving population [17,19,20].

Species are likely to experience some degree of temporal heterogeneity in nature, but its effect is likely to be dependent upon the speed of environmental fluctuations. Our data suggest that coevolution is more strongly constrained in rapidly fluctuating productivity environments, and that this occurs, at least in part, because of the relative time scales of environmental fluctuations and the dynamics of selective sweeps in victim populations. Conversely, coevolutionary interactions are likely to be intensified in more constant environments. At macroecological scales, we might therefore predict that antagonistic coevolution would be more intense in non-seasonal, tropical environments compared with seasonal environments at higher latitudes, which experience greater temporal heterogeneity [40–42]. At landscape scales, our findings have implications for the geographic mosaic theory of coevolution [3], suggesting that habitat patches with greater environmental constancy should act as coevolutionary hotspots, while patches where environmental conditions are temporally variable may be coevolutionary coldspots, depending upon the relative rates of environmental fluctuation and coevolutionary change [43,44]. A possible applied implication of this is that industrial or agricultural practices that increase environmental homogeneity could intensify coevolutionary interactions of resident species with their associated enemies.

## Data accessibility

Data deposited to DRYAD.
